# Genetic KCa3.1-Deficiency Produces Locomotor Hyperactivity and Alterations in Cerebral Monoamine Levels

**DOI:** 10.1371/journal.pone.0047744

**Published:** 2012-10-15

**Authors:** Kate Lykke Lambertsen, Jan Bert Gramsbergen, Mithula Sivasaravanaparan, Nicholas Ditzel, Linda Maria Sevelsted-Møller, Aida Oliván-Viguera, Maj Rabjerg, Heike Wulff, Ralf Köhler

**Affiliations:** 1 Department of Neurobiology Research, University of Southern Demark, Odense, Denmark; 2 Cardiovascular and Renal Research, Institute of Molecular Medicine, University of Southern Denmark, Odense, Denmark; 3 KMEB, Molecular Endocrinology, Odense University Hospital, Odense, Denmark; 4 Department of Pharmacology, University of California Davis, Davis, California, United States of America; 5 Aragon Institute of Health Sciences I+CS and ARAID, Zaragoza, Spain; University of Houston, United States of America

## Abstract

**Background:**

The calmodulin/calcium-activated K^+^ channel KCa3.1 is expressed in red and white blood cells, epithelia and endothelia, and possibly central and peripheral neurons. However, our knowledge about its contribution to neurological functions and behavior is incomplete. Here, we investigated whether genetic deficiency or pharmacological activation of KCa3.1 change behavior and cerebral monoamine levels in mice.

**Methodology/Principal Findings:**

In the open field test, KCa3.1-deficiency increased horizontal activity, as KCa3.1^−/−^ mice travelled longer distances (≈145% of KCa3.1^+/+^) and at higher speed (≈1.5-fold of KCa3.1^+/+^). Working memory in the Y-maze was reduced by KCa3.1-deficiency. Motor coordination on the rotarod and neuromuscular functions were unchanged. In KCa3.1^−/−^ mice, HPLC analysis revealed that turn-over rates of serotonin were reduced in frontal cortex, striatum and brain stem, while noradrenalin turn-over rates were increased in the frontal cortex. Dopamine turn-over rates were unaltered. Plasma catecholamine and corticosterone levels were unaltered. Intraperitoneal injections of 10 mg/kg of the KCa3.1/KCa2-activator SKA-31 reduced rearing and turning behavior in KCa3.1^+/+^ but not in KCa3.1^−/−^ mice, while 30 mg/kg SKA-31 caused strong sedation in 50% of the animals of either genotypes. KCa3.1^−/−^ mice were hyperactive (≈+60%) in their home cage and SKA-31-administration reduced nocturnal physical activity in KCa3.1^+/+^ but not in KCa3.1^−/−^ mice.

**Conclusions/Significance:**

KCa3.1-deficiency causes locomotor hyperactivity and altered monoamine levels in selected brain regions, suggesting a so far unknown functional link of KCa3.1 channels to behavior and monoaminergic neurotransmission in mice. The tranquilizing effects of low-dose SKA-31 raise the possibility to use KCa3.1/KCa2 channels as novel pharmacological targets for the treatment of neuropsychiatric hyperactivity disorders.

## Introduction

The calcium/calmodulin-activated K^+^ channel KCa3.1 [1], [2] is voltage-independent and its primary cell biological role is to produce solid membrane hyperpolarization in response to increases in intracellular calcium concentrations. KCa3.1 is widely expressed in non-excitable tissues, e.g. erythrocytes (here known as the Gardos channel) [3], [4], white blood cells [5], salivary glands [6], vascular endothelia [7], [8] and intestinal and bronchial epithelia [9]. In these tissues KCa3.1 channels contribute to cell volume regulation, migration, and proliferation and thus play a role in modulating immune responses, fibrosis, restenosis disease, blood pressure, and fluid secretion [5], [10]. Genetic deficiency of KCa3.1 in mice has been shown to produce splenomegaly (likely caused by defective erythrocyte volume regulation) [3], endothelial dysfunction and mild systolic hypertension during locomotor activity [8], [11], but no overt immunological deficits. In the brain, KCa3.1 channels are expressed in the blood brain barrier (i.e. cerebrovascular endothelium) [12] and in activated microglia [13], [14] where they are involved in respiratory burst [14], nitric oxide production and inflammatory responses in the wake of ischemic stroke and traumatic brain injury [15], [16]. Whether or not KCa3.1 is also expressed in central or peripheral neurons, is a matter of debate since the initial cloning papers reported that KCa3.1 is absent from neuronal tissue based on Northern blot analysis [1], [17], [18]. However, since then several studies reported expression of KCa3.1 in human dorsal root ganglia [19] and enteric neurons [20]. More recently, KCa3.1 expression has also been reported in cerebellar Purkinje cells of the rat in which the channel has been shown to contribute to after-hyperpolarizations and thereby regulation of excitatory postsynaptic potentials by suppressing low frequencies of parallel fiber input [21]. Despite these findings no behavioral phenotype has been reported in KCa3.1^−/−^ mice.

**Figure 1:**
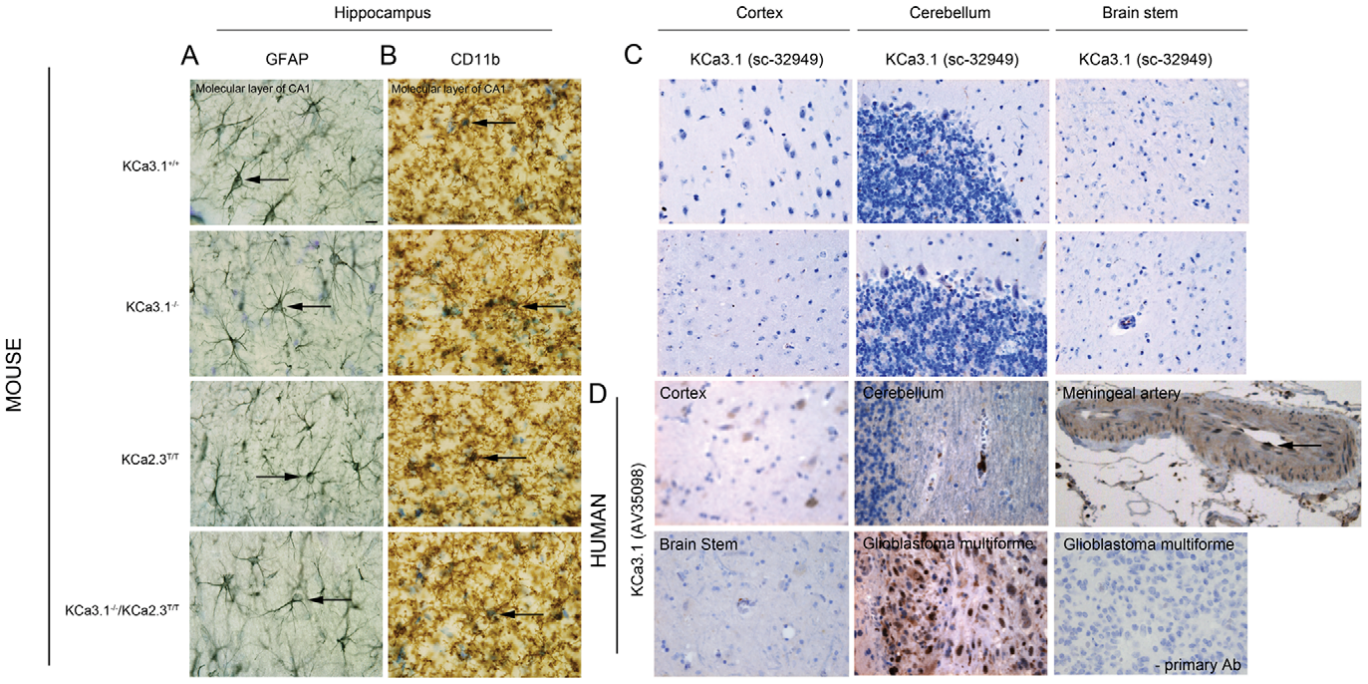
Brain morphology and immunohistochemistry. (**A**) No overt differences between genotypes in morphology and amounts of glial fibrillary acidic protein (GFAP)^+^ astrocytes and (**B**) of CD11b^+^ microglia in the molecular layer of the hippocampal CA1 region. (**C**) Immune histochemistry of KCa3.1 in brain tissues from KCa3.1^+/+^ and KCa3.1^−/−^ mice and (**D**) in human brain tissues, meningeal artery, and glioblastoma multiforme together with a negative control (−AB).

**Table 1:** Radiographic assessment of body composition of KCa3.1^−/−^ and KCa3.1^+/+^ mice.

	KCa3.1^+/+^ (n = 6)	KCa3.1^−/−^ (n = 7)	% difference of KCa3.1^−/−^ relative to KCa3.1^+/+^	P-value
**Total body mass, g**	25±1	28±2	13	**<0.01**
**Bone Mineral Density, g/c 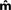 2**	0.044±0.001	0.052±0.002	16	**<0.001**
**Bone Mineral Content, g**	0.32±0.07	0.42±0.02	29	**<0.05**
**Bone Area, c 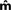 2**	7.3±0.6	8.1±0.3	11	**NS**
**% Fat tissue**	28±4	31±2	11	**NS**
**% Lean tissue**	72±4	69±2	−4	**NS**

**Table 2:** Radiographic assessment of body composition of KCa2.3^T/T^ and KCa3.1^−/−^/KCa2.3^T/T^ mice.

	KCa2.3^T/T^ (n = 11)	KCa3.1^−/−^/KCa2.3^T/T^ (n = 11)	% difference of KCa3.1^−/−^/KCa2.3^T/T^ relative to KCa2.3^T/T^	P-value
**Total body mass, g**	26±1	31±1	17	**<0.001**
**Bone Mineral Density, g/c 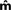 2**	0.051±0.001	0.053±0.001	2	**NS**
**Bone Mineral Content, g**	0.36±0.02	0.44±0.02	22	**<0.01**
**Bone Area, c 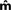 2**	7.0±0.2	8.3±0.3	19	**<0.01**
**% Fat tissue**	26±1	28±1	9	**P = 0.09**
**% Lean tissue**	74±1	72±1	−3	**NS**

**Table 3:** Radiographic assessment of hind limb composition of KCa3.1^−/−^ and KCa3.1^+/+^ mice.

	KCa3.1^+/+^ (n = 6)	KCa3.1^−/−^ (n = 7)	% difference of KCa3.1^−/−^ relative to KCa3.1^+/+^	P-value
**Hind limb mass, g**	0.60±0.04	0.66±0.05	11	**NS**
**Bone Mineral Density, g/c 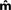 2**	0.043±0.001	0.052±0.003	23	**< 0.05**
**Bone Mineral Content, g**	0.0181±0.0010	0.0234±0.0005	29	**< 0.01**
**Bone Area, c 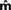 2**	0.43±0.03	0.45±0.02	6	**NS**
**% Fat tissue**	27±5	30±2	10	**NS**
**% Lean tissue**	71±6	70±3	−2	**NS**

**Table 4:** Radiographic assessment of hind limb composition of KCa2.3^T/T^and KCa3.1^−/−^/KCa2.3^T/T^ mice.

	KCa2.3^T/T^ (n = 11)	KCa3.1^−/−^/KCa2.3^T/T^ (n = 11)	% difference of KCa3.1^−/−^/KCa2.3^T/T^ relative to KCa2.3^T/T^	P-value
**Hind limb mass, g**	0.55±0.04	0.61±0.04	10	**NS**
**Bone Mineral Density, g/c 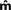 2**	0.054±0.003	0.056±0.001	4	**NS**
**Bone Mineral Content, g**	0.019±0.001	0.022±0.001	15	**P = 0.07**
**Bone Area, c 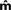 2**	0.35±0.01	0.39±0.02	12	**NS**
**% Fat tissue**	25±1	26±1	7	**NS**
**% Lean tissue**	74±3	74±2	0	**NS**

**Data in all tables are given as mean ± SEM and means were compared by unpaired Student´s T test.**

In contrast to KCa3.1, the three related KCa2.X channels (KCa2.1, KCa2.2, and KCa2.3), which exhibit a smaller unitary conductance but a similar calmodulin-dependent activation and voltage-independence, are undoubtedly present in both the soma and dendrites of central neurons [22], [23]. While KCa2.1 and KCa2.2 are most prominently expressed in the cortex and the hippocampus, KCa2.3 is enriched in subcortical areas like the striatum, thalamus and monoaminergic nuclei [24]. KCa2.X channels shape neurotransmission and firing frequency by underlying the apamin-sensitive medium after-hyperpolarization (AHP) current and have accordingly been implicated in the regulation of neuronal excitability, synaptic plasticity, learning, and memory [25], [26]. For example, mice in which KCa2.3-expression can be suppressed by insertion of a tetracycline-sensitive genetic switch exhibited working/short-term memory deficits when treated with doxycycline (DOX) and an antidepressant-like phenotype in the forced swim test [27], [28]. These changes were paralleled by enhanced dopamine and serotonin (5-HT) signaling. Similarly, mice over-expressing KCa2.2 exhibit impaired hippocampal-dependent learning and memory in both the Morris water maze and a contextual fear-conditioning paradigm, while administration of the KCa2 channel blocker apamin improves the performance of rats or mice in the water maze or in the object recognition test (for recent complete review see [24]). However, higher doses of apamin induce seizures [24].

**Figure 2:**
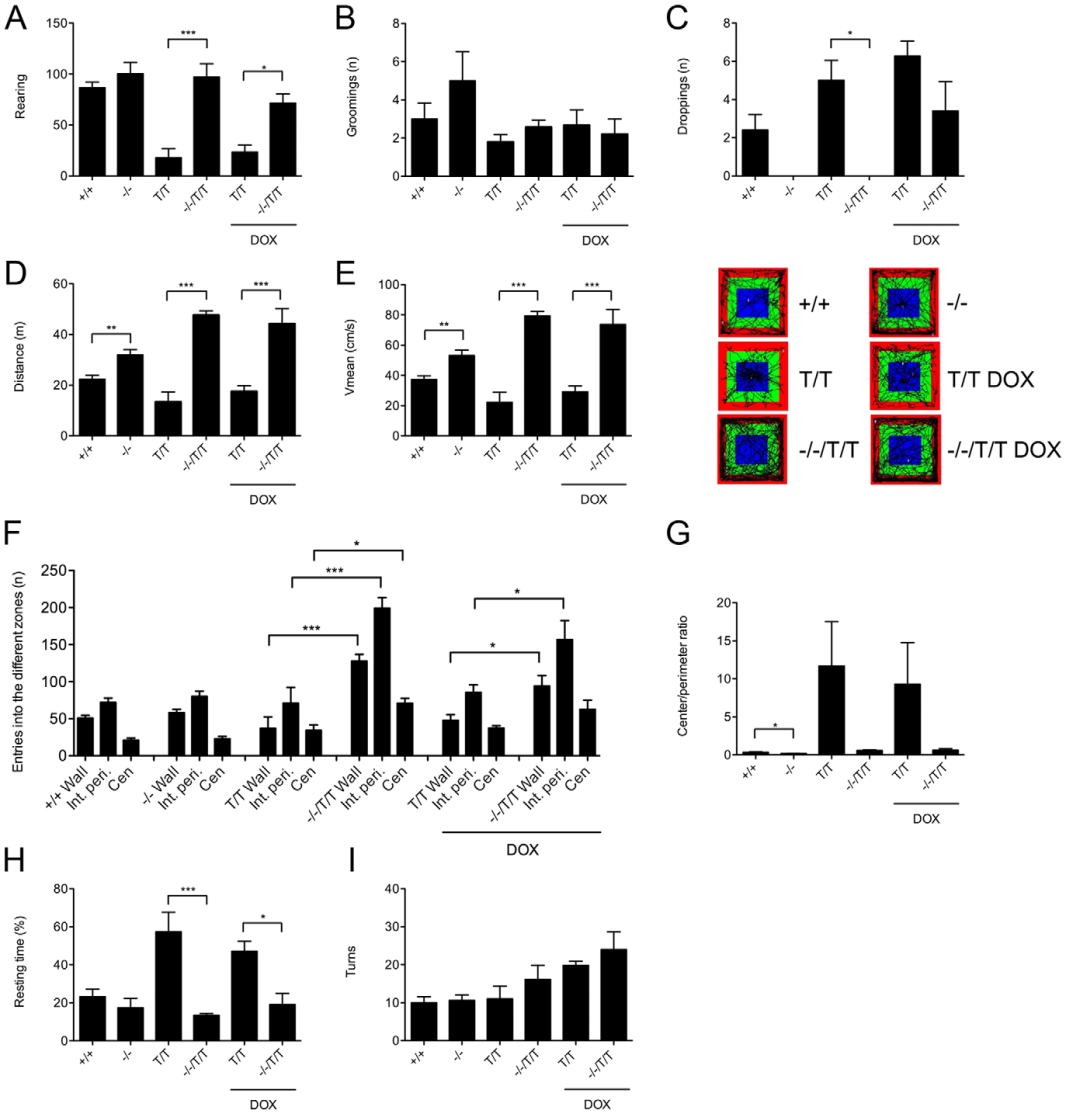
Behavioral comparisons in the open field test. Rearing activity (**A**) was similar in KCa3.1^−/−^ (^−/−^; n = 3) and KCa3.1^+/+^(^+/+^; n = 5) mice. Increased rearing behavior was observed in untreated and DOX-treated KCa3.1^−/−^/KCa2.3^T/T^ (^−/−^/^T/T^−DOX, n = 5, and +DOX, n = 5) compared to untreated and DOX-treated KCa2.3^T/T^ (^+/+^/^T/T^ (−DOX, n = 5, and +DOX, n = 7) mice. (**B, C**) Grooming behavior and droppings. (**D**) The mean total distance travelled in the open field was significantly increased in KCa3.1^−/−^ mice (n = 8) compared to KCa3.1^+/+^ mice (n = 10) and in untreated and DOX-treated KCa3.1^−/−^/KCa2.3^T/T^ (−DOX, n = 6, and +DOX, n = 5) compared to untreated and DOX-treated KCa2.3^T/T^ (−DOX, n = 5, and +DOX, n = 7) mice. (**E**) Higher mean velocity in KCa3.1^−/−^ and untreated and DOX-treated KCa3.1^−/−^/KCa2.3^T/T^ mice and representative SMART video tracking files. (**F**) The number of entries into the different zones was similar in KCa3.1^−/−^ and KCa3.1^+/+^, while untreated and DOX-treated KCa3.1^−/−^/KCa2.3^T/T^ mice displayed a higher number of entries into the different zones compared to KCa2.3^T/T^ mice. (**G**) Ratio between time spent at the margins and in the center of the arena was significantly decreased in KCa3.1^−/−^ mice compared to KCa3.1^+/+^ mice and tended to be increased in untreated and DOX-treated KCa2.3^T/T^ mice compared to KCa3.1^−/−^/KCa2.3^T/T^ mice. (**H**) Resting time was increased in untreated and DOX-treated KCa2.3^T/T^ mice. (**I**) Numbers of turns was similar in all genotypes and treatments. Data are given as means ± SEM.* P<0.05, ** P<0.01, *** P<0.001, unpaired Student's T-test (KCa3.1^−/−^ vs. KCa3.1^+/+^) and One-way ANOVA followed by Tukey's Multiple Comparison test (KCa2.3^T/T^+/−DOX vs. KCa3.1^−/−^/KCa2.3^T/T^+/−DOX). Kruskal-Wallis followed by Dunn's multiple Comparison test (in C).

While KCa2.X channels thus clearly play an important role in the CNS, next to nothing is currently known about KCa3.1 in CNS functions and behavior. One exception is the recently reported observation that KCa3.1^−/−^ mice are hyper-responsive to restrain-induced stress as concluded from an enhanced adrenocorticotropic hormone release from KCa3.1-deficient corticotrophs of the pituitary and a subsequently higher corticosterone level [29]. Our previous findings of systolic hypertension primarily during locomotor activity [8] further raise the possibility of altered sympathetic drive. In our present study, we therefore hypothesized that the KCa3.1 channel might contribute to behavioral regulation and that genetic KCa3.1 deficiency in mice or pharmacological activation by systemic application of the KCa3.1/KCa2.X activator naphtha [1,2–d] thiazol-2-ylamine (SKA-31) [30] may cause behavioral alterations. Moreover, we tested whether KCa3.1 deficiency and pharmacological activation produce alterations in peripheral and/or central monoamine levels. In addition, we sought to determine the impact of KCa3.1 deficiency on behavioral phenotypes caused by over-expression or suppression of the related KCa2.3 channel.

**Figure 3:**
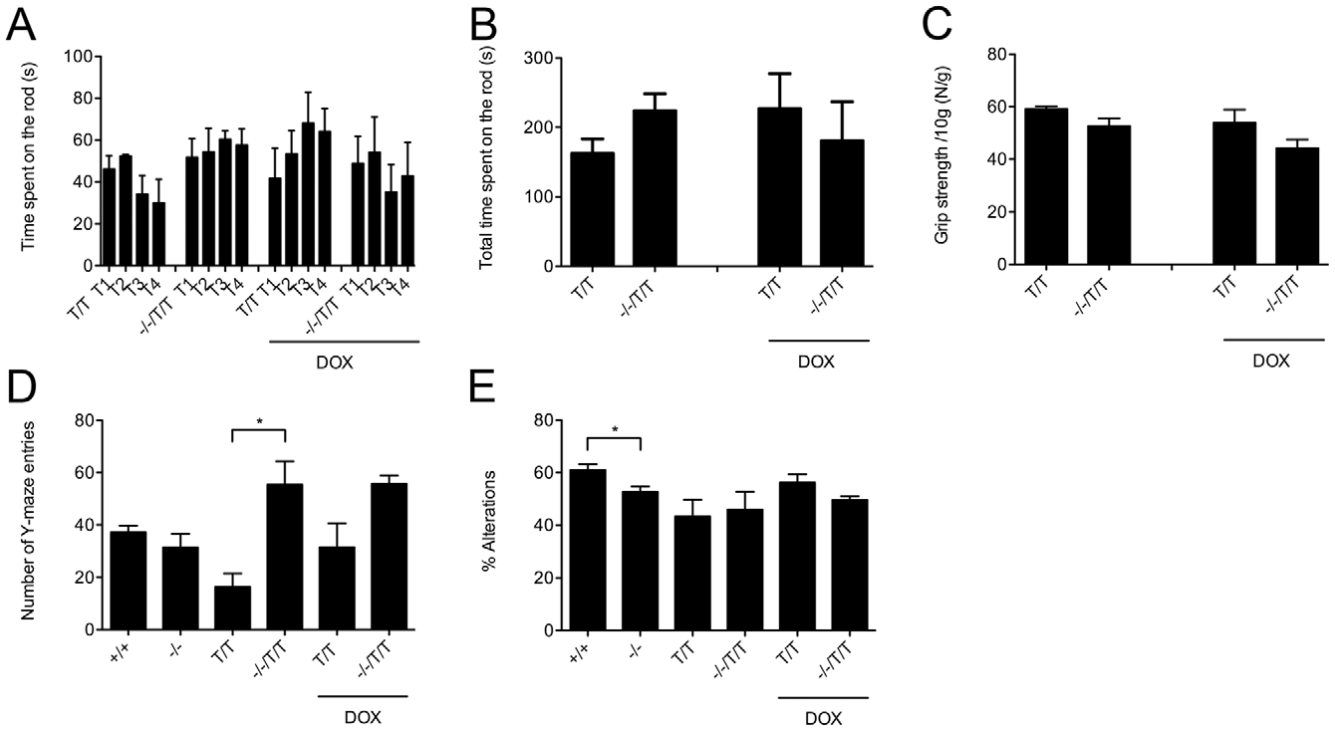
Analysis of motor coordination, grip strength, and of working memory and explorative behavior. Rotarod: (**A**) Times achieved on the accelerating rotarod in 4 consecutive trails (T1–T4); KCa2.3^T/T^ (−DOX, n = 3, and +DOX, n = 5) and KCa3.1^−/−^/KCa2.3^T/T^ (−DOX, n = 6, and +DOX, n = 5). Note that the KCa2.3^T/T^+DOX showed learning skills while this was not obvious in the other untreated or DOX-treated genotypes. (**B**) Untreated or DOX-treated genotypes spend similar total times (sum of T1–T4) on the rotarod. (**C**) Grip strengths were similar in untreated and DOX-treated genotypes; KCa2.3^T/T^ (−DOX, n = 5, and +DOX, n = 6) and KCa3.1^−/−^/KCa2.3^T/T^ (−DOX, n = 6, and +DOX, n = 5). (**D, E**) Y-maze: KCa3.1^−/−^ mice (n = 5) showed a similar number of entries (**D**) into the Y-maze but fewer alterations than KCa3.1^+/+^ mice (n = 8) (**E**). KCa2.3^T/T^−DOX (n = 6) showed significant less entries into the arms of the Y-maze but a similar number of alterations compared to KCa3.1^−/−^/KCa2.3^T/T^DOX (n = 7). The DOX-treatment did not significantly change the number of entries and alterations of KCa3.1^−/−^/KCa2.3^T/T^+DOX (n = 8) and of KCa2.3^T/T^+DOX (n = 5) mice, although KCa2.3^T/T^+DOX showed by trend a higher number of entries and alterations than KCa2.3^T/T^ mice. (D) * P<0.05, One-way ANOVA followed by Tukey's Multiple Comparison test. (E) * P<0.05, unpaired Student's T-test.

Our behavioral studies demonstrate that genetic KCa3.1 deficiency enhances locomotor activity. These behavioral alterations were paralleled by changes in central, but not peripheral monoamine levels. In contrast, the KCa3.1/KCa2.X activator SKA-31 produced hypoactivity and sedation in a dose-dependent fashion.

**Figure 4:**
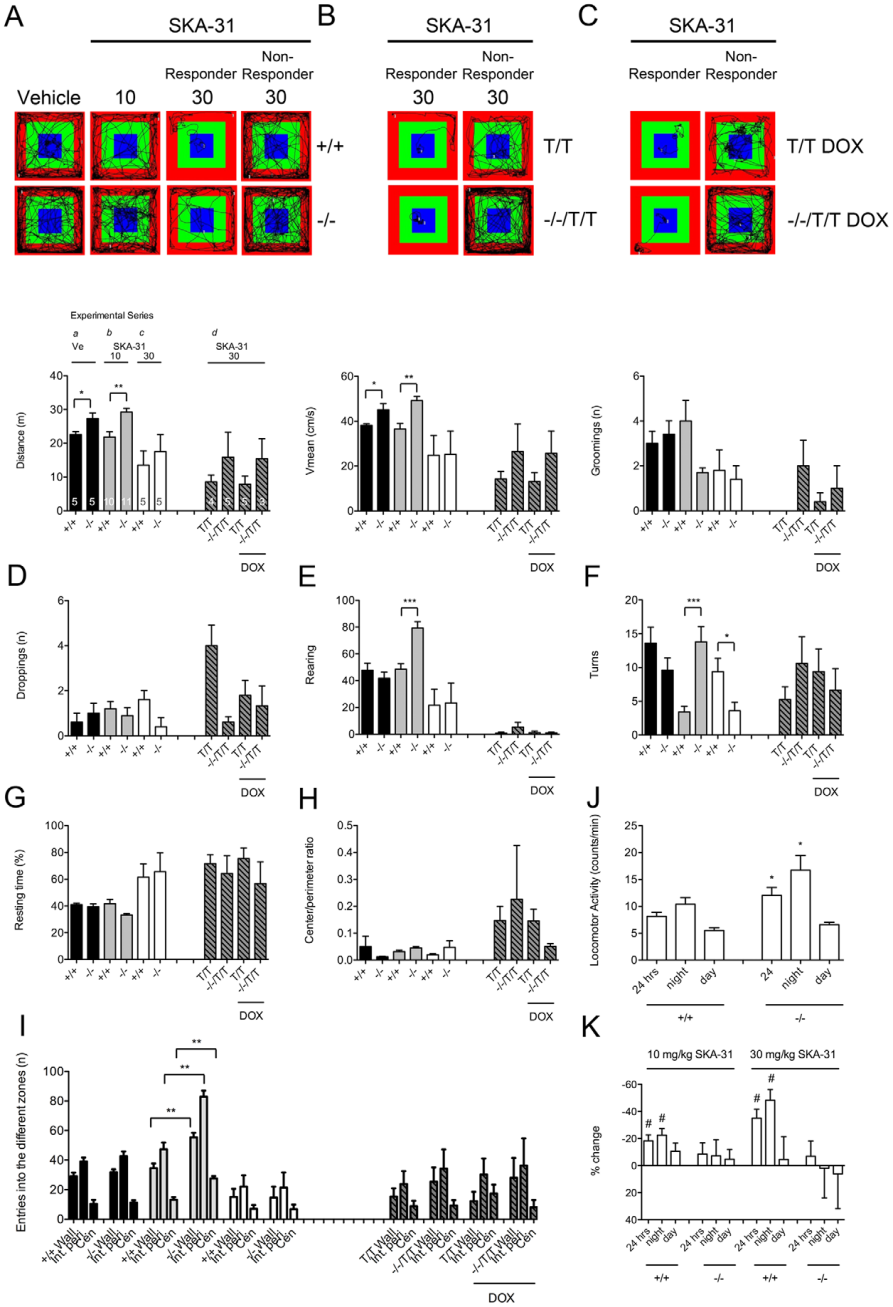
Impact of SKA-31 on locomotor activity in the open field environment and on spontaneous home cage activity in KCa3.1^−/−^ and KCa3.1^+/+^ mice. Effects of 10 mg/kg or 30 mg/kg SKA-31 (i.p.), or vehicle on distance travelled (**A**), mean velocity (**B**), grooming behavior (**C**), droppings (**D**), rearing (**E**), number of turns (**F**), resting time (**G**), center/perimeter time ratios (**H**), and entries into the different zones (**I**). In DOX-treated and untreated KCa2.3^T/T^ and KCa3.1^−^/^−^/KCa2.3^T/T^ (right part of the graphs), 30 mg/kg, SKA-31 produced similar sedation. Representative SMART video tracking files show lasting immobilization in one mouse of either genotype. Numbers in columns (A) are the numbers of mice per group. Statistical comparisons were made between the different series of experiments *(a–e)* as indicated in (A) and by different column fillings. *P<0.05; **P<0.01; unpaired Student's T-test. (**J**) 24-hrs telemetric recording showed that KCa3.1^−/−^ (n = 7) had higher diurnal home cage locomotor activity (counts/min) than KCa3.1^+/+^ (n = 8). Note that the higher 24-hrs-average activity was due to a higher activity during the dark phase (activity phase of mice). Data are given as means ± SEM. * P<0.05; unpaired Student's T-test. (**K**) SKA-31 (10 and 30 mg i.p at the end of the light phase) lowered night locomotor activity in KCa3.1^+/+^ (10 mg, n = 5; 30 mg n = 3) but not KCa3.1^−/−^ (10 mg, n = 5; 30 mg n = 3). * P<0.05, before vs. after SKA-31 injections in KCa3.1^+/+^; paired Student's T-test.

## Materials and Methods

### Ethics Statement

Human tissue collection was performed by the Department of Pathology, Odense University Hospital and approved by the ethics committee of the University of Southern Denmark. Informed consent for such stored and anonymized material was not required and we obtained a waiver (CEIC No. 11/2012 and 13/2012) from our IRB to use this material. Animal protocols were approved by the Danish authorities (Dyreforsøgstilsynet, No. 2009/561–1740).

**Figure 5:**
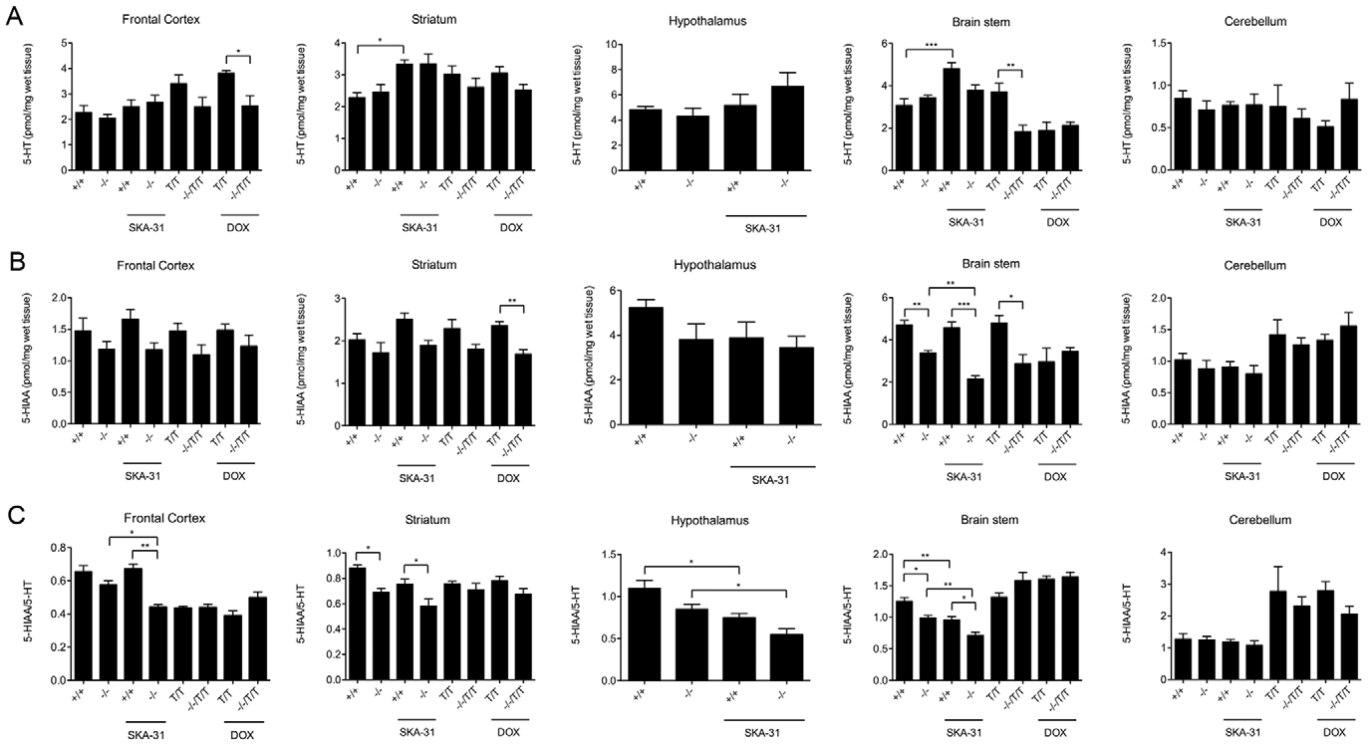
HPLC analysis of 5-HT (A) and its metabolite 5-HIAA (B), and turn-over rates (5-HIAA/5-HT) (C) in frontal cortex, striatum, hypothalamus (only KCa3.1^−/−^ and KCa3.1^+/+^), brain stem, and cerebellum. For vehicle (Ve) or SKA-31 experiments group sizes were n = 5 for KCa3.1^−/−^ and KCa3.1^+/+^, for KCa2.3^T/T^−DOX n = 5 and KCa2.3^T/T^+DOX n = 6, for KCa3.1^−/−^/KCa2.3^T/T^−DOX n = 6, and for KCa3.1^−/−^/KCa2.3^T/T^+DOX, n = 5). * P<0.05, ** P<0.01; *** P<0.001, One-way ANOVA followed by Tukey's Multiple Comparison test.

### Animals

Age- and sex-matched KCa3.1^−/−^, KCa3.1^+/+^, KCa2.3^T/T^, and KCa3.1^−/−^/KCa2.3^T/T^ mice [8], [26], [31] were derived from our breeding colonies established at the Biomedical Laboratory, University of Southern Denmark. In KCa3.1^−/−^ mice, we targeted the KCa3.1 gene by replacing exon-4 encoding for the channel pore by a neomycin-resistance cassette. Loss of KCa3.1-gene function was demonstrated by loss of KCa3.1-currents in endothelial cells and T-lymphocytes [8]. The initial homozygous mice were on a mixed 129S/SvEv x C57BL/6J background and were then backcrossed over 8 generations onto a C57BL/6J background. KCa3.1^+/+^ mice littermates were used as controls. KCa3.1^−/−^/KCa2.3^T/T^ mice were derived from crossbreeding of KCa2.3^T/T^ mice (on a mixed 129S4/SvJae x C57BL/6J background) onto the KCa3.1^−/−^ background. KCa2.3^T/T^ mice (expressing wild type (WT) levels of KCa3.1) were derived from the same breeding. In KCa2.3^T/T^ mice, the insertion of a tetracycline (T)-sensitive gene switch downstream of the endogenous promoter results in a strong 10-fold over-expression while dietary DOX treatment reduces gene expression by 90% leading to an almost complete loss of functional KCa2.3 expression [26]. All animals used for this study were derived from inbred (strain) breeding (3–4 generations) because of the double-transgenic nature of the KCa3.1^−/−^/KCa2.3^T/T^ mice. Male C57BL/6J mice were from Taconic Ltd (Ry, Denmark). Mice in all groups were 3±1 month old. Subgroups of KCa2.3^T/T^ and KCa3.1^−/−^/KCa2.3^T/T^ mice received either 2 mg/ml DOX in 2% sucrose-containing drinking water or only 2% sucrose-containing drinking water for at least 2 weeks before experimentation. For SKA-31 injections, SKA-31 was dissolved in peanut oil and injected intraperitoneally (i.p.) (injection volume: 100 µl) 30 min before starting the open field test (OFT) or within the last 30–60 min of the light phase.

**Figure 6:**
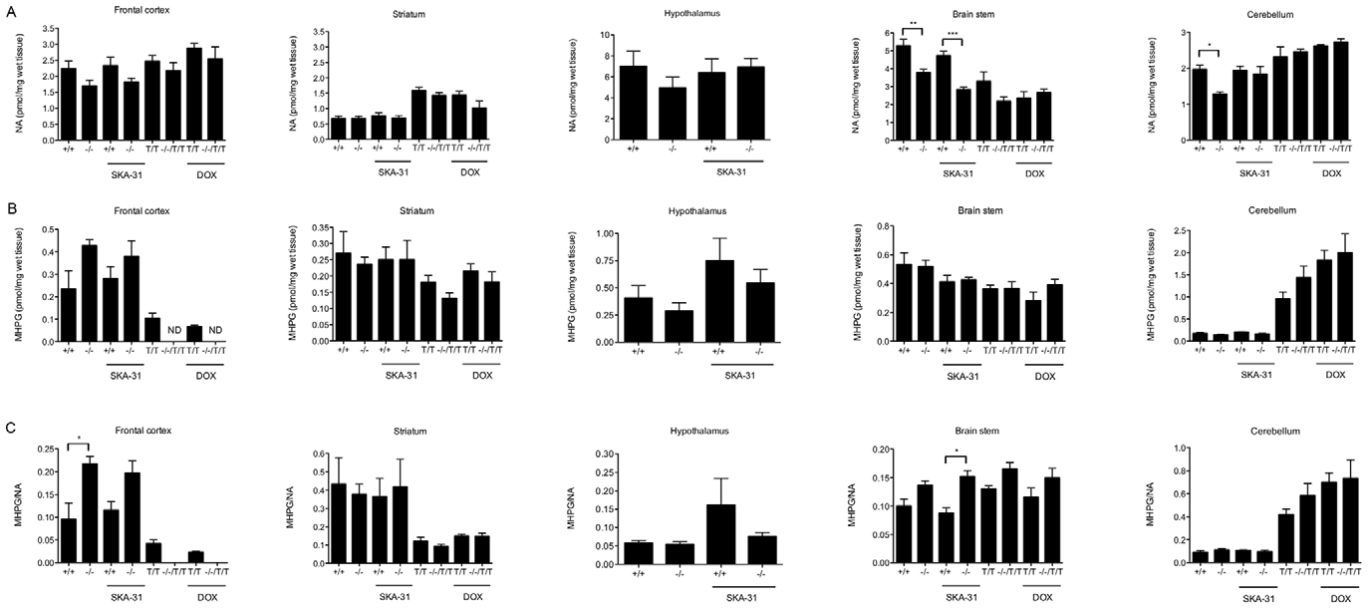
NA (A) and MHPG (B) levels in frontal cortex, striatum, hypothalamus (only KCa3.1^−/−^ and KCa3.1^+/+^), brain stem, and cerebellum. (**C**) NA turnover rates. Note that MHPG levels in the frontal cortex of KCa3.1^−/−^/KCa2.3^T/T^ were below the detection level in this analysis (ND, not detected). For group sizes (n) see legend to figure 5. * P<0.05, ** P<0.01; *** P<0.001, One-way ANOVA followed by Tukey's Multiple Comparison test.

### Behavioral tests

#### Open field test

OFT was performed with a non-transparent, squared plastic box (45×45×45 cm) over a period of 10 min. Movements were tracked using the SMART video tracking software (Panlab, Barcelona, Spain) connected to a video camera (SSC-DC378P, Biosite, Stockholm, Sweden). The distance travelled (meter), speed (cm/sec), resting time, turns, and the entries into the three zones (Wall, Inter peri and Center of the box) were recorded automatically. Rearing, grooming, and droppings were recorded manually and are given as number (n) of events [32].

**Figure 7:**
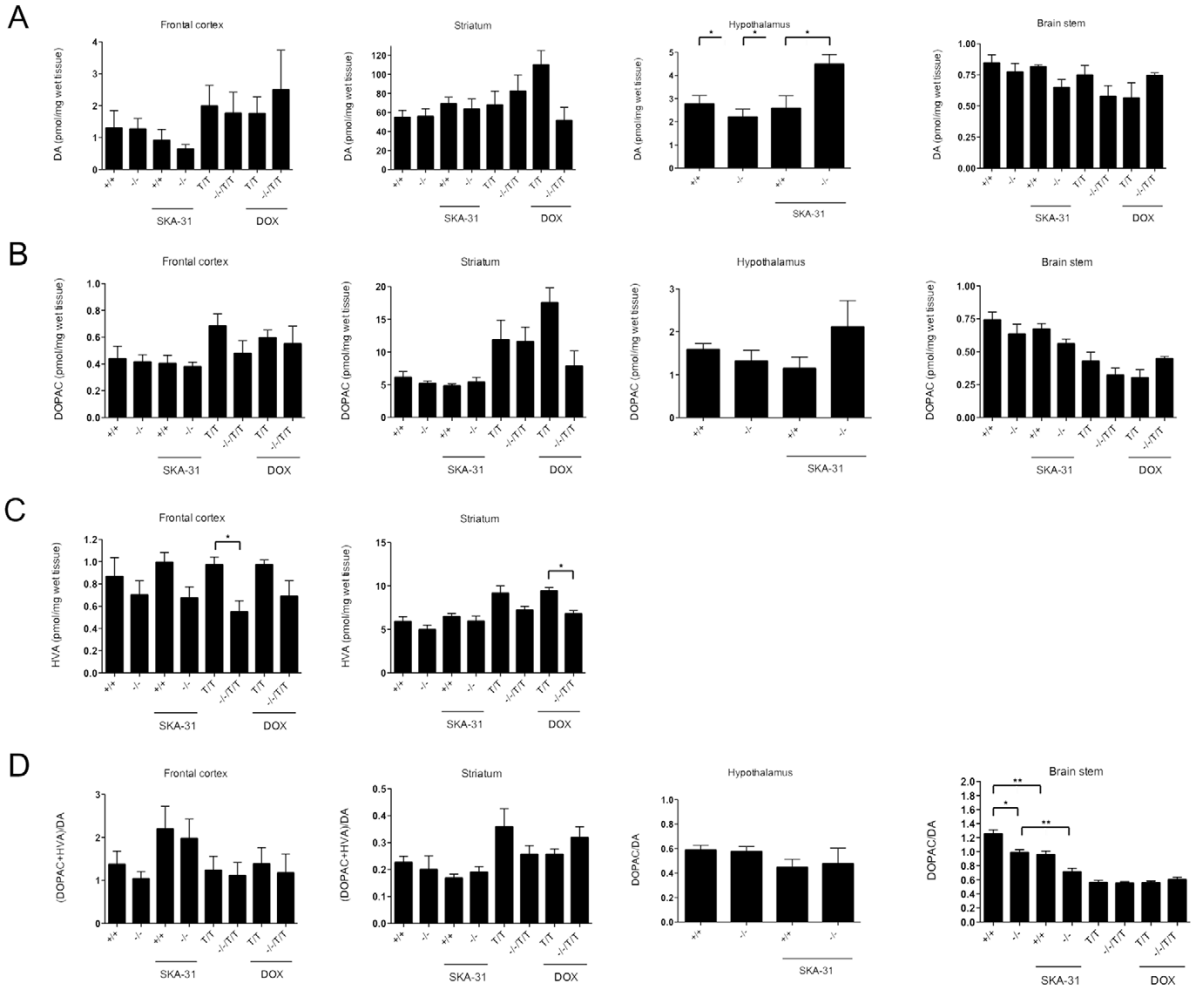
Levels of DA (A) and its metabolites DOPAC (B) in frontal cortex, striatum, hypothalamus (only KCa3.1^−/−^ and KCa3.1^+/+^), and brain stem in either genotype or treatment. For group sizes (n) see legend to figure 5. (**C**) Levels of the second DA metabolite HVA. Note that HVA levels in the hypothalamus and brain stem were below the detection level in this analysis and DA turn-over rates are given as DOPAC/DA ratios for these tissues. (**D**) DA-turnover rates. * P<0.05, ** P<0.01, One-way ANOVA followed by Tukey's Multiple Comparison test.

#### Y-maze test

Spontaneous alteration behavior and hence working memory was evaluated in KCa3.1^+/+^ and KCa3.1^−/−^, DOX-treated and untreated KCa2.3^T/T^ and KCa3.1^−/−^/KCa2.3^T/T^ mice using the Y-maze (arm length: 40 cm, arm bottom width: 7 cm, arm upper width: 8 cm, height of the wall: 16 cm). Each mouse was placed in the arm designated (a) of the Y-maze field. The number of entries, except for the first two, and alterations were recorded manually. Data were collected for 8 min.

**Figure 8:**
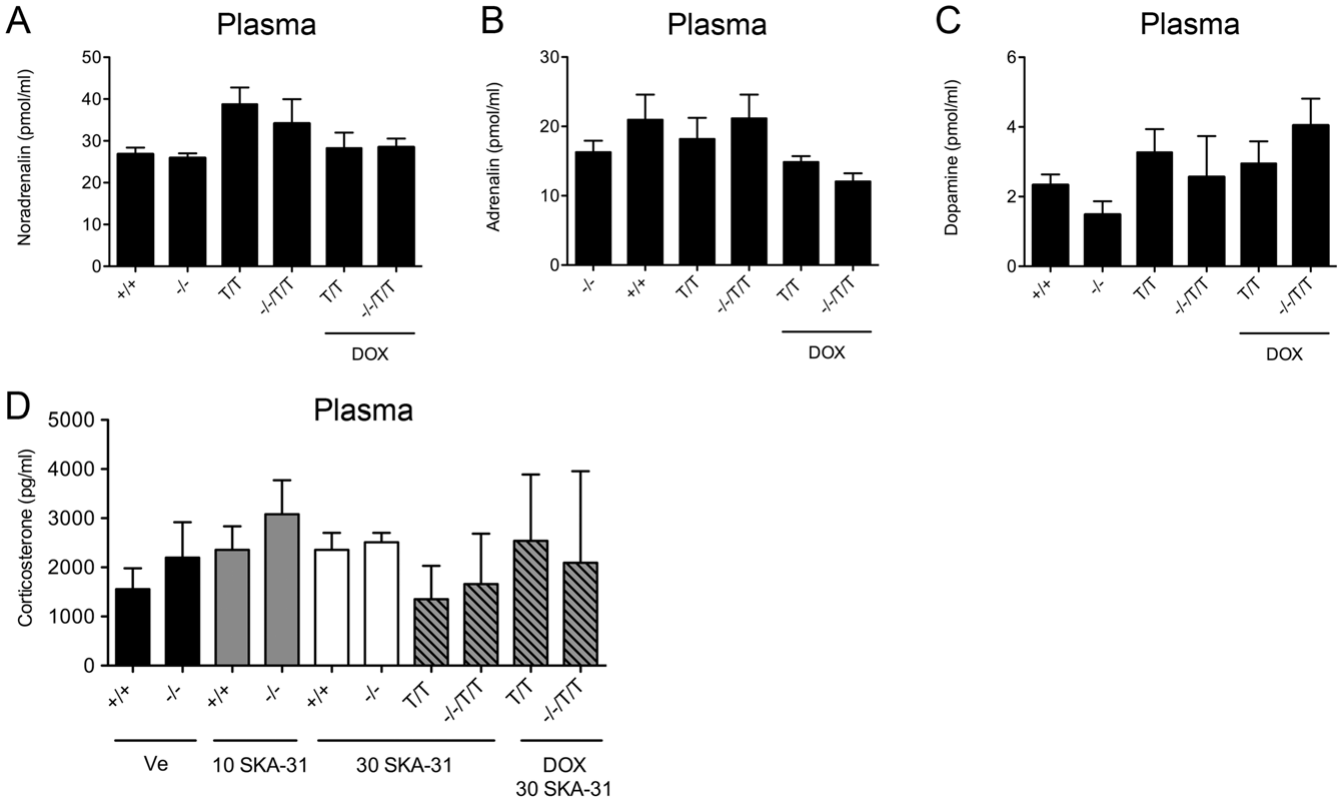
Plasma catecholamine and corticosterone **levels.** Levels of NA, adrenalin (ADR), and DA in plasma from KCa3.1^+/+^ and KCa3.1^−/−^ (for NA and ADR n = 12 per genotype; for DA: n = 7 (KCa3.1^+/+^) and n = 5 (KCa3.1^−/−^) and in plasma from KCa2.3^T/T^(−DOX, n = 5, and +DOX, n = 5) and KCa3.1^−/−^/KCa2.3^T/T^ (−DOX, n = 5, and +DOX, n = 5). Plasma corticosterone levels were unchanged in all genotypes and treatments. Ve: n = 4 (KCa3.1^+/+^) and n = 5 (KCa3.1^−/−^; for SKA-31: n = 5 (KCa3.1^+/+^) and n = 5 (KCa3.1^−/−^); KCa2.3^T/T^, n = 3 (−DOX), n = 2 (+DOX); KCa3.1^−/−^/KCa2.3^T/T^, n = 5 (−DOX), n = 3 (+DOX).

#### Grip strength test

The grip strength meter (BIO-GT-3, BIOSEB, Sweden) was used to study the neuromuscular function by determining the maximum force required for KCa2.3^T/T^, KCa2.3^T/T^+DOX, KCa3.1^−/−^/KCa2.3^T/T^ and KCa3.1^−/−^/KCa2.3^T/T^+DOX mice to release their grips. Mice were allowed to grasp the metal grid and then pulled backwards in the horizontal plane. The force applied to the grip was recorded as the peak tension. Total (both) front paw strengths were measured. Each mouse was tested in 5 sequential trials and the highest grip strength was recorded as the score [33].

#### The rotarod test

Was performed using a LE8200 system (Panlab Harvard Apparatus). The full test consisted of two parts; pre-training and 4 test trials (T1 – T4) [34], [35]. *Pre-training*: mice were pre-trained to stay and walk on the rod for 30 seconds at 4 rounds per minute (rpm). Mice that were not able to do this were excluded from the subsequent test. All KCa3.1^−/−^/KCa2.3^T/T^ and KCa3.1^−/−^/KCa2.3^T/T^+DOX mice fulfilled the criteria for the rotarod test, whereas 2 KCa2.3^T/T^ and 2 KCa2.3^T/T^+DOX mice were excluded from the test due to failure of fulfilling the criteria in the pre-training. *Trial*: Prior to testing, the mice were allowed to acclimatize in the behavior room for at least 1 hour. Mice were placed on the rotarod and speed of the rotor was accelerated from 4 to 40 rpm over 5 minutes. The latency to fall from the rod was recorded automatically. Each mouse was tested in 4 trials with 20 min resting time between each trial.

### Tissue processing for histology

Mice were given an overdose of pentobarbital and perfused through the left ventricle using 20 ml chilled 4% paraformaldehyde (PFA) in 0.15 M phosphate-buffered saline, pH 7.4 (Lambertsen et al, 2009). Brains were quickly removed and post-fixated in 4% PFA for 1 hour and either immersed in 20% sucrose overnight, frozen and sectioned into a series of 8 parallel, 20 μm thick cryostat sections, which were stored at −80°C until further processing. Other brains were transferred to 1% PFA followed by 0.1% PFA, before serial cutting into 60 μm thick sections, which were stored in de Olmos cryoprotective solution at −12°C until further processing. One series of sections from each mouse was stained with Toluidine blue for visualization of the general histology [36].

### Immunohistochemistry

Microglial CD11b and astroglial glial fibrillary acidic protein (GFAP) were visualized in vibratome sections using a three-step biotin-streptavidin-horseradish peroxidase (HRP) technique as described in detail elsewhere [36]. KCa3.1 and KCa2.3 immunohistochemistry (IHC) was performed on sections from paraffin-embedded brain tissues. Sections were dewaxed with xylene, rehydrated through an alcohol gradient, treated with 1.5% H_2_O_2_, demasked with T-EG buffer, and incubated for 1 hour with primary antibody in antibody diluent S2022. The following primary antibodies (ABs) against KCa3.1 were used: Santa Cruz sc-32949 (1∶500), Alomone ALM-051 (1∶2000), and Sigma AV35098 (1∶2000) followed by EnVision+polymer K4003(rabbit)/K4001(mouse). The ABs ALM-051 (1∶2000) and Sigma AV35098 gave similar results (not shown). Sections were visualized with DAB and buffered Substrate Kit (Dako K3468), counterstained with hematoxylin and mounted with Aquatex. A second set of staining with a different antigen retrieval protocol, a different secondary antibody and the Vectastain kit was performed independently and gave similar results [15]. For IHC studies on KCa3.1 protein expression in human post mortem brain material, paraffin-embedded tissue was kindly provided by the Department of Pathology, Odense University Hospital and processed in the same way. As negative control we processed sections omitting the primary antibody (AV35098 against KCa3.1) or by substituting the primary antibody with rat IgG2a isotype control (CD11b) or rabbit serum (GFAP) as previously described [36].

### High performance liquid chromatography analysis of monoamines in plasma and brain samples and corticosterone measurements

Mice were scarified by decapitation and blood (about 300 µl) was collected in Eppendorf vials containing EDTA, centrifuged (1,000 g for 10 min, 4°C) and the plasma (about 150 µl per sample) was stored at −20°C until further processing. Prior to high performance liquid chromatography (HPLC) analysis, plasma catecholamines were extracted using the ClinRep® complete kit (Recipe GmbH, Munich, Germany).

After decapitation, the brain was quickly removed from the skull and different brain regions were rapidly dissected, placed on dry ice, weighed, and stored at −80°C. At the day of HPLC analysis, the brain tissue samples were briefly sonicated in Eppendorf vials containing 200–1000 µl (about 1∶20 w/v) of 0.1 M perchloric acid (PCA) with antioxidants (0.2 g/l Na2S2O5, 0.05 g/l Na2-EDTA) and centrifuged at 20,627 g for 20 minutes at 4°C. The supernatant was used for HPLC analysis.

Levels of noradrenalin (NA), dopamine (DA), serotonin (5-hydroxytryptamine, 5-HT) as well as the DA metabolites 3,4-dihydroxyphenylacetic acid (DOPAC) and homovannilic acid (HVA), the NA metabolite 3-methoxy-4-hydroxyphenylglycol (MHPG), and the 5-HT metabolite 5-hydroxyindoleacetic acid (5-HIAA) were assessed by reverse-phase HPLC with electrochemical detection, essentially as described previously [37], but using a mobile phase consisting of 10% methanol (v/v), 20 g/l citric acid monohydrate, 100 mg/l octane-1-sulfonic acid sodium salt, 40 mg/l EDTA dissolved in Milli-Q water and pH adjusted to 4.0 [38]. The Merck-Hitachi HPLC system consisted of a L-7100 pump a L-7200 autosampler, a D-7000 interface, and an electrochemical detector with in-built column oven (Decade, Antec, Leyden, Netherlands), connected to a computer equipped with D-7000 version 2.0 chromatography software. The mobile phase was pumped at a flow rate of 0.9 ml/min through a Waters Spherisorb S5 ODS2 guard column (4.6×30 mm) and a Waters Spherisorb S3 ODS2 cartridge analytical column (4.6×150 mm, Waters, MA, USA). A mixture of external standards was injected to identify and quantify the compounds of interest.

Plasma corticosterone levels were determined in mice sacrificed immediately after OFT and 40–45 minutes after treatment with either vehicle or 30 mg/kg SKA-31. Corticosterone was detected in 10 µl plasma samples (diluted 1∶12) and quantified using the DetectX®Corticosterone CLIA Kit (Arbor Assays, Ann Arbor, Michigan) according to manufacturer's instructions.

### Telemetry

Telemetric recording of voluntary diurnal home cage activity and analysis of data was performed after implanting a TA11PAC10 transducer (Data Sciences International (DSI), St Paul, Minnesota, USA) as described previously [8].

### Body composition measurements

Total tissue mass (g), fat mass (g), fat-%, lean tissue mass (g), lean-%, bone area (cm^2^), bone mineral content (BMC, g), and bone mineral density (BMD, g/cm^2^) were measured using dual-energy X-ray absorptiometry (DXA) using a PIXImus2 (Version 1.44; Lunar Corporation, Madison, WI, USA).

### Statistics

Data are given as means ± SEM. Data sets for corresponding strains (KCa3.1^−/−^ vs. KCa3.1^+/+^) and treatments (KCa3.1^−/−^/KCa2.3^T/T^ vs. KCa2.3^T/T^; KCa3.1^−/−^/KCa2.3^T/T^+DOX vs. KCa2.3^T/T^+DOX, and untreated strains vs. DOX-treated strains) were compared using unpaired or paired (SKA-31 treatments) two-tailed Student's T test if appropriate and as indicated in the legend text. For testing differences in droppings we used the non-parametric Kruskal-Wallis followed by Dunn's multiple Comparison test because of unequal variances in the groups. For multiple comparisons of multiple data sets within one or of more than two groups, we used One-way ANOVA followed by Tukey's Multiple Comparison test, if appropriate and as indicated in the figure legends. Statistical analysis was performed using Prism 5 software for Mac OS X, GraphPad Software. Statistical significance was established for P<0.05.

## Results

### Body composition of KCa3.1^−/−^, KCa3.1^+/+^, KCa2.3^T/T^, and KCa3.1^−/−^/KCa2.3^T/T^ mice

Body weight was significantly higher in male KCa3.1^−/−^ mice (28.3±0.8 g, n = 20) than in male KCa3.1^+/+^ mice (23.5±0.7 g, n = 17; P<0.001), but similar in female KCa3.1^−/−^ mice (20.4±0.4 g, n = 15) and female KCa3.1^+/+^ mice (19.4±0.7 g, n = 19; P = 0.28). Also, male KCa3.1^−/−^/KCa2.3^T/T^ mice (27.9 g±0.6 g, n = 11) displayed a higher body weight compared to male KCa2.3^T/T^ mice (24.8±0.7 g, n = 11; P<0.01), while body weights were again similar in female KCa3.1^−/−^/KCa2.3^T/T^ mice (18.5±0.5 g, n = 10) and female KCa2.3^T/T^ mice (19.6±0.9 g, n = 9; P = 0.31). We found no morphological alterations of major organs with the exception of mild splenomegaly in KCa3.1^−/−^ mice and KCa3.1^−/−^/KCa2.3^T/T^ mice as reported previously [8]. Our radiographic assessment of total body mass, % lean mass, % fat mass, bone mineral density (BMD) and bone mineral content (BMC) (Tables 1 and 2) as well as of the same parameters in the hind limb (Tables 3 and 4) revealed no major differences between genotypes, with the exception of higher total body masses of male KCa3.1^−/−^ mice (+3 g) compared to male KCa3-1^+/+^ mice (Table 1) and of male KCa3.1^−/−^/KCa2.3^T/T^ mice (+5 g) compared to male KCa2.3^T/T^ mice (Table 2) and a higher BMD and BMC in male KCa3.1^−/−^ mice compared to male KCa3.1^+/+^ mice (Table 1). General brain morphology and structures of major brain regions showed no abnormalities in any of the strains based on toluidine blue staining (data not shown). Immunohistochemical staining for GFAP^+^ astrocytes and CD11b^+^ microglia revealed no apparent differences in glial number or morphology in either genotype (Figure 1A and B). Similar results were independently obtained when staining for IBA^+^ microglia (data not shown). These results suggested that life-long genetic deficiency of KCa3.1 or over-expression/suppression (using DOX) of KCa2.3 do not result in gross alterations of brain morphology. Specific immunoreactivity for KCa3.1 was not detectable in neurons, astrocytes, or microglia from any brain area of either genotype suggesting that KCa3.1 is not expressed at immunohistochemically detectable levels in the normal murine CNS (Figure 1C). Similar observations were previously made in rat brain [15] and in our current study in human brain tissues, that were found to lack KCa3.1-immunoreactivity (Figure 1D). In contrast, positive KCa3.1 immunoreactivity was observed in murine and human tissues such as endothelial cells (of human meningeal artery in the present study) and glioblastoma multiforme (Figure 1D), which were previously shown to express KCa3.1 [7], [8], [39], [40].

### Evaluation of behavioral phenotype in KCa3.1^−/−^ mice

We investigated whether the lack of KCa3.1 could lead to alterations in activity patterns, explorative behavior and/or emotionality/fear in the open field environment (Figure 2). We found that KCa3.1^−/−^ and KCa3.1^+/+^ mice were similar in their rearing and grooming behavior (Figures 2A and 2B). KCa3.1^+/+^ mice produced fecal pellets (droppings) while none of the KCa3.1^−/−^ did this in this test (Figure 2C). This difference did however not reach statistical significance (P = 0.1). Interestingly, KCa3.1^−/−^ mice travelled a ≈1.5-fold longer distance than the KCa3.1^+/+^ mice (P<0.01) (Figure 2D) and did this at a significantly higher mean velocity in all zones (≈1.4-fold, P<0.01) (Figure 2E). Despite the increased mean velocity and total distance travelled, the number of entries into the different zones was similar in KCa3.1^−/−^ and KCa3.1^+/+^ mice (P = 0.36) (Figure 2F). The ratio of center time/perimeter time for the KCa3.1^−/−^ mice was significantly smaller (≈50%) compared to the ratio for KCa3.1^+/+^ mice (Figure 1G) (P<0.05). Resting times (Figure 2H) as well as the numbers of turns were comparable between the two genotypes (P = 0.36 and P = 0.78, respectively; Figure 2I). To exclude that some of these significant differences were simply caused by an abnormal low activity level in wild-type littermate controls (KCa3.1^+/+^), we compared KCa3.1^+/+^ mice (backcrossed onto C57BL/6J background over 8 generations) with commercially available C57BL/6J mice and found that C57BL/6J mice behaved similarly to KCa3.1^+/+^ mice in the open field environment (Figure S1). Gender or body mass did not seem to have an influence on these behavioral parameters studied since KCa3.1^−/−^ females with a body mass comparable to KCa3.1^+/+^ females behaved similar to KCa3.1^−/−^ males with a higher body mass compared to KCa3.1^+/+^ males (not shown).

In KCa2.3^T/T^ or KCa3.1^−/−^/KCa2.3^T/T^ mice, over-expression of KCa2.3 or acute suppression of KCa2.3 by DOX treatment did not change behavior in the open field environment (Figure 2). However, KCa2.3^T/T^ reared 5-times less than KCa3.1^−/−^/KCa2.3^T/T^ (P<0.01) (Figure 2A), revealing substantial lower vertical explorative activity in these mice. Grooming behavior was found to be similar (P = 0.18; Figure 2B). However, the total number of droppings was significantly increased in KCa2.3^T/T^ compared to the KCa3.1^−/−^/KCa2.3^T/T^ (P = 0.004; Figure 2C). The total distance travelled in the open field box was shorter in the KCa2.3^T/T^ mice while KCa3.1^−/−^/KCa2.3^T/T^ mice covered a 3-fold longer distance (P<0.0001; Figure 2D). Similar to the difference between KCa3.1^−/−^ and KCa3.1^+/+^ mice, the high horizontal activity in KCa3.1^−/−^/KCa2.3^T/T^ mice was paralleled by a 3-fold higher velocity in all zones compared to the low velocity of KCa2.3^T/T^ mice (Figure 2E) (P<0.001). Moreover, KCa3.1^−/−^/KCa2.3^T/T^ mice showed an increased number of entries into the wall, perimeter, and center zones compared to KCa2.3^T/T^ mice (P<0.05 and P<0.001; Figure 2F). Even though there was a clear trend, the ratio of center time/perimeter time for KCa3.2^T/T^ mice was not statistically different from that of KCa3.1^−/−^/KCa2.3^T/T^ mice (P = 0.064; Figure 2G). Resting time was significantly higher in the KCa2.3^T/T^ mice than in the KCa3.1^−/−^/KCa2.3^T/T^ mice (P<0.001; Figure 2H). The number of turns did not differ between KCa2.3^T/T^ and KCa3.1^−/−^/KCa2.3^T/T^ mice (P = 0.3; Figure 2I). Taken together, the results from the open field analysis of the different genotypes demonstrate that KCa3.1-deficiency induces hyperactivity.

### Analysis of motor coordination

KCa2.3 channels are expressed in dopaminergic neurons of the substantia nigra [22], [26] and the cerebellum [24], [26], [41] while KCa3.1 channels have recently been reported to be present in rat cerebellar neurons [21]. We therefore tested whether untreated or DOX-treated KCa2.3^T/T^ and KCa3.1^−/−^/KCa2.3^T/T^ mice displayed alterations in motor coordination using the rotarod test (Figure 3). The latencies to fall from the rotating rod in four consecutive trials did not differ between groups and all four groups spent similar total times on the rotarod, suggesting that expression changes in KCa3.1 and KCa2.3 do not have any overt effect on motor coordination (Figure 3A, B).

### Analysis of muscle force

We also studied whether the higher physical activity of KCa3.1^−/−^ mice was paralleled by increased muscle force, using the grip strength test (Figure 3C). However, a major difference in muscle force was not apparent, as grip strengths were comparable in all groups. Nonetheless, there was a trend towards slightly stronger grip strengths in untreated KCa2.3^T/T^ mice compared to untreated KCa3.1^−/−^/KCa2.3^T/T^ mice (P = 0.09). DOX treatment did not alter muscle force in DOX-treated KCa2.3^T/T^ mice when compared to untreated KCa2.3^T/T^ mice (P = 0.34). However, there was a trend for DOX treatment to reduce the grip strength in KCa3.1^−/−^/KCa2.3^T/T^ mice (Figure 3C), even though the difference did not reach statistical significance (P = 0.09).

### Analysis of working memory in KCa3.1^−/−^ mice

To examine working memory, we performed the Y-maze test in KCa3.1^+/+^, KCa3.1^−/−^, KCa3.1^−/−^/KCa2.3^T/T^ and KCa2.3^T/T^ mice (Figure 3D and 3E). In this test, one can judge on working memory by counting whether an animal remembers whether it entered one arm of the Y-maze before and then explores the other arm next (alteration). KCa3.1^−/−^ and KCa3.1^+/+^ mice displayed similar numbers of entries into each arm of the Y-maze (Figure 3D). Alteration rate, however, was slightly decreased in KCa3.1^−/−^ mice compared to KCa3.1^+/+^ mice (P<0.05; Figure 3E). When comparing KCa2.3^T/T^ mice and KCa3.1^−/−^/KCa2.3^T/T^ mice, the number of entries was strongly decreased in KCa2.3^T/T^ mice (P<0.01; Figure 3D). In fact, 3 of 6 KCa2.3^T/T^ mice had less than 20 entries and alterations rate ranged from 0% to 70%. However, after exclusion of the KCa3.2^T/T^ mice with less than 20 entries, alteration rates appeared normal in the KCa3.2^T/T^ mice (P = 0.81) while DOX-treated KCa3.1^−/−^/KCa3.2^T/T^ mice showed a trend towards decreased alteration rates compared to DOX-treated KCa3.2^T/T^ (P = 0.065; Figure 3E). These results suggested that working memory was mildly impaired by KCa3.1-deficiency but not by KCa2.3^T/T^ over-expression or suppression. KCa2.3^T/T^ mice again displayed a decreased exploratory activity that could be rescued by KCa3.1-deficiency. DOX-treatment did not influence the performances of KCa3.2^T/T^ and KCa3.1^−/−^/KCa3.2^T/T^ mice (Figure 3D and E).

### Behavioral impact of the KCa3.1-activator SKA-31 in the open field environment and the effect of KCa3.1-deficiency and SKA-31 on home cage locomotor activity

Since KCa3.1-deficiency produced hyperactivity, we hypothesized that pharmacological activation of KCa3.1/KCa2.X channels by SKA-31 produces hypoactivity (Figure 4). At a dose of 10 mg/kg administered intraperitoneally 30 min prior to the open field test, SKA-31 did not produce any overt differences in the total distance travelled (Figure 4A) or the mean velocity (Figure 4B) of KCa3.1^−/−^ and KCa3.1^+/+^ mice. Grooming behavior and the number of droppings were also unaffected (Figure 4C and 4D). In contrast, SKA-31 produced low vertical explorative behavior (rearing) in the KCa3.1^+/+^ mice but not in KCa3.1^−/−^ mice (P<0.001; Figure 4E). Moreover, SKA-31-treated KCa3.1^+/+^ mice made fewer turns than SKA-31-treated KCa3.1^−/−^ mice (P<0.001; Figure 4F). Resting time, center/perimeter time ratios and entries into the different zones were unchanged by the treatment (Figure 4G–I). At the higher dose of 30 mg/kg, SKA-31 produced visible and lasting immobilization in 3 of 5 of the mice in both genotypes (see representative tracings in Figure 4 and for all tracings Figure S2), affecting all locomotor activity parameters (Figure 4A–I), although if compared to vehicle-treated mice the differences did not reach statistical significance because two mice in each group did not respond to 30 mg/kg SKA-31 and produced high variance within groups. In DOX-treated and untreated KCa2.3^T/T^ and KCa3.1^−/−^/KCa2.3^T/T^, 30 mg/kg, SKA-31 sedated 2 or 3 mice of each group (n = 3–5, see Figure S2 for all tracings) and e.g. rearing was nearly abolished (see right part of the graphs in Figure 4A–I). After 90 minutes the sedated mice recovered and freely travelled in the open field environment (see tracings in Figure S2, bottom panel).

Because of the higher locomotor activity in the open field environment, we were interested in further defining spontaneous hyperactivity in KCa3.1^−/−^ mice over longer time intervals. Moreover, we wanted to know whether pharmacological channel activation by SKA-31 in the presence of KCa3.1 had long-lasting activity lowering effects. The 24-hours telemetric recording of voluntary diurnal home cage activity (Figure 4J) revealed that the average 24-hours-activity was higher in KCa3.1^−/−^ mice than in KCa3.1^+/+^ mice (≈+47%, P<0.05). The increased activity level was apparent during the normal activity phase, i.e. the dark phase, (≈+61%, P<0.05) but less obvious during the resting “light” phase (≈+20%, P = 0.15). Pharmacological activation of KCa3.1 in KCa3.1^+/+^ gave the opposite results (Figure 4K). Injections of 10 and 30 mg/kg (i.p.) of SKA-31 into KCa3.1^+/+^ mice at the end of the light phase decreased activity over 24 hours by ≈−16% (P<0.01) and ≈−36% (P = 0.06), respectively, an effect being most apparent during the dark phase (10 mg/kg: ≈−22%, P<0.01; 30 mg/kg: ≈−49%, P<0.05). During the second 12 hrs period of the light phase the effect was smaller (10 mg/kg: −5%, P = 0.14; 30 mg/kg: −8%, P = 0.17), which can be explained by decreasing plasma levels due to metabolism of the compound [30]. This activity lowering effect of SKA-31 was not seen in KCa3.1^−/−^ mice (Figure 4J–K).

These results suggested that SKA-31 produced low vertical and home cage activity levels over at least 12 hours in a KCa3.1-dependent manner, while the strong immobilization caused by the high dose in some of the mice did not depend on KCa3.1 or expression levels of KCa2.3 and is most likely due to the activation of other KCa2 subtypes.

### Central monoamine levels in KCa3.1^−/−^, KCa2.3^T/T^, and KCa3.1^−/−^/KCa2.3^T/T^ mice and after treatment with SKA-31

HPLC analysis of several major brain areas revealed that KCa3.1-deficiency did not alter 5-HT levels (Figure 5A) in the frontal cortex, striatum, hypothalamus, cerebellum, and brain stem of vehicle-treated KCa3.1^−/−^ compared to vehicle-treated KCa3.1^+/+^ mice. However, SKA-31 (30 mg/kg) significantly increased 5-HT levels in the striatum and brain stem of KCa3.1^+/+^ mice and also by trend in the striatum from KCa3.1^−/−^ mice. SKA-31 did not change 5-HT levels in frontal cortex, hypothalamus, and cerebellum in either group.

In KCa2.3^T/T^+DOX mice, 5-HT-levels were increased in the frontal cortex (+34%; P<0.01) while untreated KCa2.3^T/T^ mice showed a trend towards increased levels (+27%; P = 0.1) compared to KCa3.1^−/−^/KCa2.3^T/T^+DOX mice and untreated KCa3.1^−/−^/KCa2.3^T/T^ mice, respectively. In the striatum, 5-HT tended to be higher in KCa2.3^T/T^+DOX and untreated KCa2.3^T/T^ mice compared to KCa3.1^−/−^/KCa2.3^T/T^+DOX mice. In untreated KCa2.3^T/T^ mice, brain stem levels of 5-HT were higher than in KCa2.3^T/T^+DOX mice, untreated KCa3.1^−/−^/KCa2.3^T/T^ mice, and KCa3.1^−/−^/KCa2.3^T/T^+DOX mice.

Levels of the 5-HT-metabolite, 5-hydroxyindoleacetic acid (5-HIAA) (Figure 5B) were lower in the brain stem of KCa3.1^−/−^ mice (P<0.01) and by trend in the hypothalamus (P = 0.11) compared to KCa3.1^+/+^ mice. SKA-31 (30 mg/kg) further reduced 5-HIAA levels in the brain stem of KCa3.1^−/−^ but not in KCa3.1^+/+^ mice. 5-HIAA was lower in the striatum (P<0.01) of DOX-treated KCa3.1^−/−^/KCa2.3^T/T^ mice and by trend in untreated KCa3.1^−/−^/KCa2.3^T/T^+DOX mice (P = 0.1) compared to DOX-treated KCa2.3^T/T^ mice and untreated KCa2.3^T/T^+DOX mice, respectively. 5-HIAA levels were not significantly different in the other brain regions studied. Calculated turn-over rates (5-HIAA/5-HT, Figure 5C) were significantly reduced in the striatum (P<0.05), brain stem (P<0.05), and by trend in the frontal cortex (P<0.1) of KCa3.1^−/−^ mice compared to KCa3.1^+/+^ mice and SKA-31 further decreased turn-over rates in the frontal cortex of KCa3.1^−/−^ mice (P<0.05) and in the hypothalamus (P<0.05) and brain stem (P<0.01) of KCa3.1^−/−^ and KCa3.1^+/+^ mice (Figure 5C). 5-HT turn-over rates (5-HIAA/5-HT) were unchanged in KCa2.3^T/T^ and KCa3.1^−/−^/KCa2.3^T/T^ mice (see right part of the graphs in Figure 5C data).

In the analyzed forebrain areas, noradrenalin (NA) levels were not changed by either genotype or treatment (Figure 6A). However, vehicle-treated and SKA-31-treated KCa3.1^−/−^ mice showed significantly reduced NA levels in the brain stem compared to vehicle-treated and SKA-31-treated KCa3.1^+/+^ mice (P<0.01; Figure 6A). In addition, vehicle-treated KCa3.1^−/−^ mice had lower NA levels in the cerebellum compared to vehicle-treated KCa3.1^+/+^ mice. NA levels in the brain stem and cerebellum of untreated or DOX-treated KCa3.1^−/−^/KCa2.3^T/T^ and KCa2.3^T/T^ mice were unaltered. Levels of the major NA metabolite 3-methoxy-4-hydroxyphenylglycol (MHPG) (Figure 6B) were not changed significantly by KCa3.1^−/−^ deficiency or treatments in any brain region. However, calculated NA turn-over rate (MHPG/NA ratio) was higher in the frontal cortex of vehicle-treated KCa3.1^−/−^ mice (P<0.05) and by trend in SKA-31-treated KCa3.1^−/−^ mice (P = 0.1) compared to vehicle-treated and SKA-31-treated KCa3.1^+/+^ mice, respectively. NA turn-over rate was also higher in the brain stem of SKA-31-treated KCa3.1^−/−^ mice (P<0.05) and by trend of vehicle-treated KCa3.1^−/−^ mice (P = 0.1) compared to vehicle-treated and SKA-31-treated KCa3.1^+/+^ mice, respectively. We did not find significant changes in NA turn-over rates in untreated or DOX-treated KCa3.1^−/−^/KCa2.3^T/T^ and KCa2.3^T/T^ mice. But, interestingly, MHPG levels were higher in the cerebellum of the KCa2.3^T/T^ and KCa3.1^−/−^/KCa2.3^T/T^ mice compared to KCa3.1^−/−^ and KCa3.1^+/+^ mice.

Dopamine (DA) levels were unaltered by genotype, the SKA-31 treatment, or the DOX treatment in the striatum, frontal cortex, and brain stem, (Figure 7A), with the only exception of a higher DA level in the hypothalamus of SKA-31-treated KCa3.1^−/−^ mice compared to any of the other groups (P<0.05). Also levels of the dopamine-metabolite 3,4-dihydroxyphenylacetic acid (DOPAC) were unaltered by genotype or treatments (Figure 7B). The other DA metabolites homovannilic acid (HVA) was found at detectable levels in the frontal cortex and striatum, but not in brain stem or hypothalamus (Figure 7C). HVA levels were not significantly changed in the frontal cortex and striatum of KCa3.1^−/−^ mice compared to KCa3.1^+/+^ mice (P = 0.46 and P = 0.25, respectively). Also after the SKA-31 treatment, HVA levels were not significantly different in these mice. In contrast, significantly lower levels of HVA were found in the frontal cortex of untreated KCa3.1^−/−^/KCa2.3^T/T^ (P<0.05) and by trend in the frontal cortex of DOX-treated KCa3.1^−/−^/KCa2.3^T/T^ DOX (P = 0.07) compared to untreated KCa2.3^T/T^ and DOX-treated KCa2.3^T/T^+DOX mice, respectively. HVA levels were significantly lower in the striatum of DOX-treated and untreated KCa3.1^−/−^/KCa2.3^T/T^ mice compared to the respective treated or untreated KCa2.3^T/T^ mice (P<0.05). Despite these differences, DA turn-over rates (DOPAC+HVA/DA ratio) in the frontal cortex and striatum were unaltered by genotype, the SKA-31 treatment, or the DOX treatment (Figure 7D). In contrast, the DOPAC/DA ratio was significantly lower in the brain stem of KCa3.1^−/−^ mice compared to KCa3.1^+/+^ mice (P<0.05) and the SKA-31 treatment reduced DOPAC/DA ratios in both genotypes (P<0.01). DOPAC/DA ratios were similar in untreated or DOX-treated KCa3.1^−/−^/KCa2.3^T/T^ and untreated or DOX-treated KCa2.3^T/T^ mice. In the hypothalamus, DOPAC/DA ratios were similar in KCa3.1^−/−^ and KCa3.1^+/+^ mice as well as after the SKA-31 treatment in these mice.

GABA levels were not altered in KCa3.1^−/−^ mice or by the SKA-31 treatment (Figure S3).

### Circulating catecholamine and corticosterone levels in KCa3.1^−/−^, KCa2.3^T/T^, and KCa3.1^−/−^/KCa2.3^T/T^ mice

Plasma catecholamine levels (NA, adrenalin (ADR), DA) were assessed at the day of sacrifice and were found not to differ between KCa3.1^−/−^ and KCa3.1^+/+^ mice as well as between KCa3.1^−/−^/KCa2.3^T/T^ and KCa2.3^T/T^ mice with or without DOX-treatment (Figure 8). However, it appeared that untreated KCa2.3^T/T^ mice had higher NA levels than KCa3.1^−/−^ (P = 0.002) and KCa3.1^+/+^ mice (P = 0.012). Plasma corticosterone levels were not statistically altered in either strain or by treatments (Figure 8).

## Discussion

Our study suggests a previously unrecognized but significant role of the KCa3.1 channel in the control of behavior and central monoaminergic transmission. We concluded this based on the following findings: 1. Genetic deficiency of KCa3.1 significantly increased locomotor activity without impairing neuromuscular functions or body physics. Locomotor hyperactivity due to KCa3.1-deficiency overcompensated for the locomotor hypoactivity normally observed in KCa2.3^T/T^ mice. 2. KCa3.1-deficiency produced slightly impaired working/short term memory. 3. Plasma catecholamine or corticosterone levels were not altered by KCa3.1 deficiency. 4. In contrast, central monoaminergic transmission was altered in several ways by KCa3.1 deficiency. KCa3.1^−/−^ mice exhibited significantly reduced turn-over rates of 5-HT in frontal cortex, striatum, and brain stem. Moreover, KCa3.1^−/−^ mice had reduced NA levels in brain stem and cerebellum and exhibited increased NA turn-over rates in the brain stem. 5. Administration of the selective KCa3.1/KCa2.X-activator SKA-31 had opposite effects on behavior as it reduced rearing, turning behavior, and home cage activity in a KCa3.1-dependent fashion and at a high dosages produced strong sedation in a KCa3.1/KCa2.3 independent manner, most likely related to KCa2.2 activation. These data suggested that KCa3.1-deficient mice show a type of attention-deficit hyperactivity disorder (ADHD) phenotype, which is related to lower 5-HT turn-over and lower NA levels in specifically the brain stem, but is not related to alterations in sympathetic drive and chronic distress. The efficacy of a selective KCa3.1/KCa2.X activator at reducing locomotor activity proposed KCa3.1/KCa2.X-channels as novel therapeutic targets for the treatment of human neurological syndromes characterized by physical hyperactivity such as ADHD.

Considering possible mechanisms underlying the behavioral alterations, our HPLC analysis of plasma and regional cerebral monoamine levels revealed normal plasma catecholamine levels, but several, brain-region specific changes in monoamine levels and turnover rates in KCa3.1^−/−^ mice, including reduced NA levels and increased NA turn-over rate in the brain stem and frontal cortex as well as reduced 5-HT turnover rates in the striatum and brain stem. Particularly, the higher NA turn-over – pointing to increased NA transmission – may be linked to the observed hyperactivity in the KCa3.1^−/−^ mice. However, whether these changes are causally related to the observed hyperactivity, remains unclear. A common finding in animal models of ADHD is hyperdopaminergic function (increased DA turnover)(for review see [42]), but this is apparently not the case in the KCa3.1-deficient mice. However, because of cross-talk between the monoaminergic systems, changes in NA and 5-HT transmission can affect dopaminergic function and ADHD symptoms. Changes in noradrenergic transmission and action on inhibitory alpha-2 autoreceptors can either enhance or ameliorate ADHD symptoms. 5-HT may play a role in hyperactivity, either directly through 5-HT_2_ receptors or indirectly by modulating dopaminergic transmission. Decreased activity of 5-HT has been reported in relation to impulsivity in borderline personality, aggression and suicide, while increased 5-HT activity and lower NA activity have been associated with ADHD (for references see [43]). However, changes in NA and/or 5-HT metabolism observed in clinical conditions or animal models may not be directly – causally – linked to hyperactivity, but rather reflect compensatory changes to counteract hyperactivity or dysfunction of other monoaminergic systems.

Recently, another KCa3.1^−/−^ mouse has been reported to be hyper-responsive to stress due to increased release of adrenocorticotropic hormone (ACTH) and corticosterone, with no change in basal corticosterone levels [29]. We likewise did not observe higher plasma corticosterone or plasma levels of catecholamine suggesting that the hyperactivity of our KCa3.1-deficient mice is not related to higher chronic distress or overt sympathetic rush.

Unlike KCa3.1-deficient mice, KCa2.3^T/T^ mice exhibited significantly higher 5-HT levels in the frontal cortex and tended to also exhibit higher 5-HIAA levels in striatum with no change in 5-HIAA and 5-HIAA/5-HT turn-over rates. These increases, which are contrary to the decreases seen in KCa3.1^−/−^ mice, could represent a compensatory mechanism to mitigate the overt hypoactivity in these animals. Higher 5-HT levels have been reported for this strain earlier [28] and the present study also showed that these alterations are presumably “developmental” as 2-week DOX-treatment and the resulting channel suppression had no impact on the 5-HT levels in these mice. It is, however, noteworthy that additional life-long KCa3.1-deficiency reduced levels to those measured in wild type mice.

While KCa3.1-deficiency produced hyperactivity, the brain penetrable KCa3.1/KCa2.X-activator SKA-31, with a 5-fold higher selectivity for KCa3.1 over KCa2.X channels, had opposite effects and produced hypoactivity and sedation in half of the animals, which at the higher dose was largely KCa3.1- and KCa2.3 independent as it occurred in KCa3.1^−/−^ and KCa2.3^T/T^ with or without DOX treatment alike. This can be explained by activation of KCa2.1 and KCa2.2 channels with a more widespread expression in the murine brain [22] and is in line with findings that higher doses of SKA-31 are anticonvulsant presumably due to KCa2.2 activation [30] and acutely improve motor deficits in SCA3 mice, a model of spinocerebellar ataxia 3 [44]. Nonetheless, at a lower dose of SKA-31 the reduction of rearing and turning behavior depended on KCa3.1. Despite this poor KCa3.1-dependence of the acute SKA-31-effects, SKA-31 had a “calming” effect on spontaneous home cage activity in the wild-type, which was not observed in KCa3.1^−/−^ mice, as expected. The acute treatment with the high dose of SKA-31 significantly increased 5-HT in the striatum and brain stem of wild type, but not in KCa3.1^−/−^ mice, and significantly reduced 5-HIAA/5-HT turn-over rates in brain stem and hypothalamus in a KCa3.1-independent fashion, which suggested that these SKA-31-effects are caused by activation of KCa2.X channels and involved a reduced 5-HT release as reflected by the lower 5-HT turn-over rate.

The mechanistic link between deficiency of KCa3.1 to the behavioral alterations and the changes of cerebral monoamine levels remains unclear. But we speculate that these alterations could be related to the endothelial dysfunction (severely impaired acetylcholine-induced vasodilation) and systolic hypertension during locomotor activity in the KCa3.1^−/−^ mice [11], [31]. In this regard KCa3.1^−/−^ mice are similar to spontaneously hypertensive rats (SHR), which are also hyperactive, show memory deficits and alterations of monoaminergic neurotransmission, and thus neurological features similar to ADHD [42]. In contrast to our model, however, 5-HT levels are increased in several brain regions in the SHR model of ADHD. On the other hand, KCa3.1^−/−^ (this study) and SHR [45] appear to have a concordant higher central NA activity.

In order to validate KCa3.1 deficient mice as an alternative model of ADHD, the behavioral effects of chronic methylphenidate treatment should be investigated in further studies.

In conclusion, the present study revealed a novel mechanistic link of the non-neuronal KCa3.1 channel to behavior, central monoamine levels, and hyperactivity in mice. From the clinical perspective, our pharmacological studies suggested that small molecule activators of KCa3.1 may have beneficial “calming” effects in hyperactivity disorders, including ADHD.

## Supporting Information

Figure S1 **Similar performances of KCa3.1^−/−^ and C57Bl6J in the open field test.** (**A**) Center/perimeter ratio, (**B**) total distance travelled, (**C**) mean velocity, (**D**) entries into the different zones. Data are given as means ± SEM.
(TIF)

Figure S2 **Original tracings of all open field test experiments.**
(TIF)

Figure S3 **GABA levels in the different brain areas from KCa3.1^−/−^ (−/−) and KCa3.1^+/+^ (+/+).** Data are given as means ± SEM.
(TIF)
