# Supplementary figures and images for: Genetic KCa3.1-Deficiency Produces Locomotor Hyperactivity and Alterations in Cerebral Monoamine Levels
Source: PLoS One. 2012 Oct 15;7(10):e47744. doi: 10.1371/journal.pone.0047744 (PMC3471871; doi:10.1371/journal.pone.0047744)

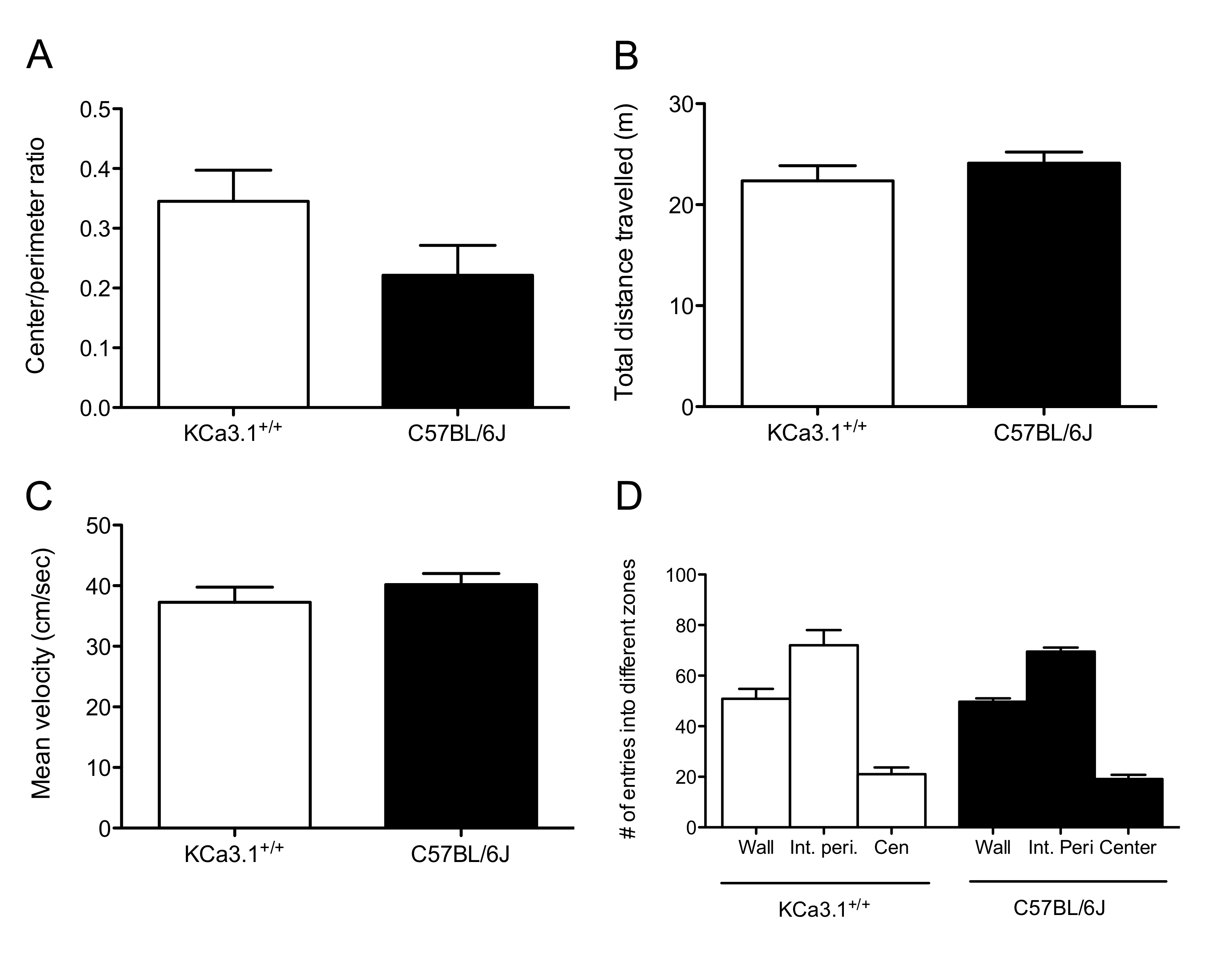

Supplement: Figure S1 — Similar performances of KCa3.1−/− and C57Bl6J in the open field test. (A) Center/perimeter ratio, (B) total distance travelled, (C) mean velocity, (D) entries into the different zones. Data are given as means ± SEM. (TIF) [file pone.0047744.s001.tif]

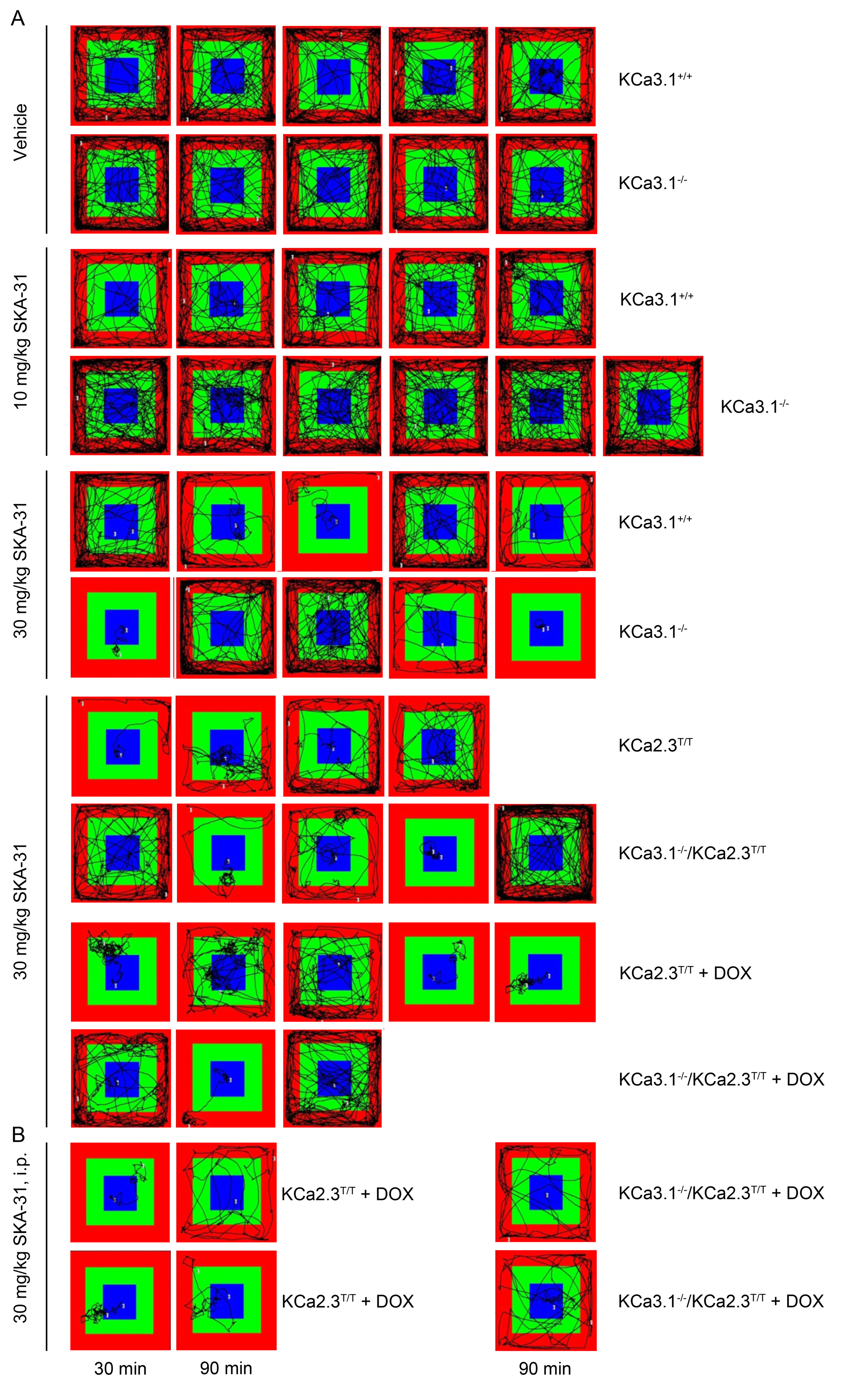

Supplement: Figure S2 — Original tracings of all open field test experiments. (TIF) [file pone.0047744.s002.tif]

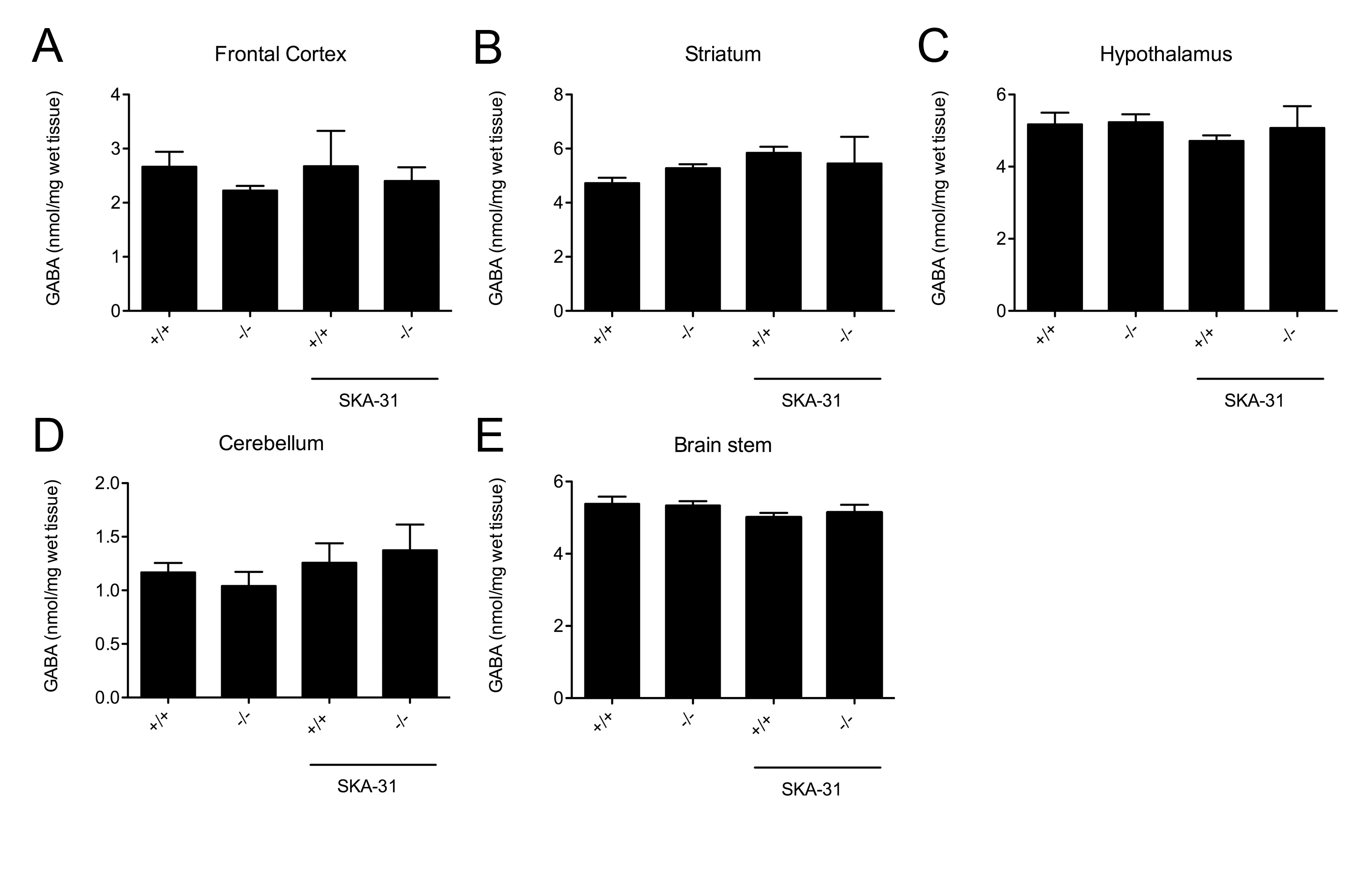

Supplement: Figure S3 — GABA levels in the different brain areas from KCa3.1−/− (−/−) and KCa3.1+/+ (+/+). Data are given as means ± SEM. (TIF) [file pone.0047744.s003.tif]
